# Spike Protein at the Crossroads of Long-COVID and Vaccine-Induced Immune Thrombotic Thrombocytopenia Syndromes: A Cardio-Hematological Perspective

**DOI:** 10.3390/biomedicines14092090

**Published:** 2026-09-17

**Authors:** Elifcan Aladag, Ugur Nadir Karakulak, Ibrahim Celalettin Haznedaroglu

**Affiliations:** 1 Hematology Department, Faculty of Medicine, Hacettepe University, Ankara 06800, Turkey; elifcan.aladag@hacettepe.edu.tr (E.A.); haznedar@gmail.com (I.C.H.); 2 Cardiology Department, Faculty of Medicine, Hacettepe University, Ankara 06800, Turkey

**Keywords:** SARS-CoV-2 spike protein, Long-COVID, endothelial dysfunction, platelet activation

## Abstract

The severe acute respiratory syndrome coronavirus 2 (SARS-CoV-2) spike (S) protein is central to viral infectivity, host–pathogen interactions, and immune recognition. Beyond its indispensable role in viral entry, accumulating experimental and clinical evidence indicates that spike protein may exert pleiotropic biological effects involving endothelial, cardiovascular, hematological, neurological, and immunological pathways. During natural infection, these effects occur in the context of active viral replication, additional viral antigens, and systemic inflammation, whereas vaccination induces a fundamentally different, transient exposure to a prefusion-stabilized spike antigen without viral propagation. In this review, we critically examine the molecular and clinicopathological properties of the SARS-CoV-2 spike protein and the biological differences between infection-derived and vaccine-derived spike exposure. Particular emphasis is placed on endothelial dysfunction, platelet and complement activation, immune-thrombosis, persistent viral antigens and tissue reservoirs in Long-COVID, and the distinct anti-PF4-mediated pathophysiology of vaccine-induced immune thrombotic thrombocytopenia (VITT). Emerging evidence suggests that persistent or dysregulated spike-related antigen exposure may contribute to chronic multisystem manifestations in a subset of individuals following SARS-CoV-2 infection; however, evidence linking persistent vaccine-derived spike to chronic clinical syndromes remains substantially more limited and does not currently establish causality. Integrating these observations, we propose the ‘Long-Spike’ hypothesis as a hypothesis-generating conceptual framework rather than a defined clinical syndrome. Further prospective studies integrating ultrasensitive antigen detection, tissue-based analyses, immunophenotyping, and cardiovascular and hematological biomarkers are required to determine the clinical relevance and causal significance of persistent spike-related antigens.

## 1. Molecular Structure of the SARS-CoV-2 Spike Protein and Its Origin

The SARS-CoV-2 spike protein is a class I viral fusion glycoprotein forming homotrimers on the viral surface. Each monomer consists of two functional subunits: S1, responsible for receptor binding, and S2, which mediates membrane fusion. The receptor-binding domain (RBD) within S1 engages angiotensin-converting enzyme 2 (ACE2), while host proteases such as TMPRSS2 and furin facilitate cleavage at the S1/S2 and S2′ sites, enabling viral entry [1]. These structural and proteolytic features not only determine viral entry and cellular tropism but may also influence the biological activity, tissue distribution, and pathogenic potential of spike-derived products.

A distinguishing molecular feature of SARS-CoV-2 compared with closely related sarbecoviruses is the presence of a polybasic furin cleavage site at the S1/S2 junction. This motif enhances viral entry and may influence tissue tropism and pathogenicity by facilitating proteolytic activation of spike and promoting cell–cell fusion and syncytium formation. Experimental disruption of this multibasic cleavage site markedly reduces spike-mediated cell–cell fusion and viral entry into human lung cells, highlighting the functional importance of furin-dependent spike processing [2]. Structural stabilization of the prefusion conformation, glycosylation patterns, and conformational dynamics collectively shape spike immunogenicity and biological activity [3].

From a clinical and pathobiological perspective, the functional consequences of spike structure are particularly relevant to disease phenotypes. Notably, the biological context of spike exposure differs substantially between natural infection and vaccination, including differences in molecular configuration, duration and magnitude of expression, cellular distribution, and the surrounding inflammatory environment.

## 2. Clinicopathological Aspects of the Spike Protein Itself

Beyond serving as a viral attachment protein, spike exhibits intrinsic biological activity. Soluble spike and S1 subunits have been detected in the circulation during acute SARS-CoV-2 infection and, transiently, following mRNA vaccination [4,5]. Spike binding to ACE2 can perturb the renin–angiotensin–aldosterone system (RAAS), shifting the balance toward angiotensin II–mediated vasoconstriction, inflammation, oxidative stress, and thrombosis.

Experimental studies indicate that spike protein can activate endothelial cells, increase vascular permeability, and induce proinflammatory signaling independent of full viral replication [6]. In addition, spike exposure has been shown to activate integrin α5β1/NF-κB signaling in endothelial cells, promoting the expression of adhesion molecules, proinflammatory cytokines, and coagulation factors while increasing endothelial permeability [7]. These properties position spike as a potential pathogenic mediator contributing to microvascular injury and immunothrombosis [8].

However, the translational relevance of these experimental observations requires caution. Many mechanistic studies have used recombinant spike or S1 protein in isolated cell systems or animal models, sometimes at concentrations substantially higher than those detected in the human circulation. For example, Perico et al. exposed human microvascular endothelial cells to S1 concentrations ranging from 0.5 to 50 nM (approximately 37.5–3750 ng/mL), with 10 nM (approximately 750 ng/mL) used for several endothelial, complement, and platelet experiments [9]. In contrast, circulating spike detected in post-acute human studies using ultrasensitive assays, including Swank et al., was measured in the picogram-per-milliliter range [10]. This substantial concentration gap limits direct extrapolation from experimental systems to human disease. It therefore remains uncertain whether circulating spike concentrations observed in vivo are sufficient, by themselves, to reproduce the endothelial and platelet effects demonstrated under experimental conditions. Local tissue concentrations, molecular form of the antigen, duration of exposure, and the surrounding inflammatory milieu may nevertheless differ from plasma measurements and could modify biological activity. Accordingly, these experimental findings should be interpreted as evidence of biological plausibility rather than proof that circulating spike at clinically observed concentrations directly causes vascular pathology.

## 3. COVID-19 Process Inducing Natural Spike Protein in the Human Body

During natural infection, the spike protein is synthesized within infected host cells as part of the viral replication cycle. Viral particles, infected cell debris, and spike-derived antigens may enter the systemic circulation, particularly in severe disease. Indeed, SARS-CoV-2 RNAemia and viremia have been demonstrated in patients with acute COVID-19 and are more frequently detected at higher levels in severe and critically ill patients [11].

The magnitude and duration of spike exposure in infection depend on viral load, host immune competence, age, and comorbidities. Importantly, infection-derived spike is accompanied by a broad array of viral proteins and pathogen-associated molecular patterns, generating a complex inflammatory milieu that amplifies tissue injury. Consistent with this concept, detectable plasma viral RNA has been associated with higher circulating inflammatory markers, including C-reactive protein and interleukin-6, as well as increased disease severity and mortality [12]. Thus, the biological effects attributed to spike during natural infection should be interpreted within the broader context of active viral replication and systemic host inflammatory responses.

## 4. Vaccines Inducing mRNA-Spike Synthesis in the Human Body

mRNA vaccines deliver nucleoside-modified mRNA encoding a prefusion-stabilized full-length spike protein, encapsulated in lipid nanoparticles [13]. Following intramuscular injection, host cells transiently express spike protein, which is presented to the immune system via major histocompatibility complex pathways and induces both humoral and cellular immune responses [14].

The vaccine-encoded spike differs structurally from the native viral spike primarily through prefusion-stabilizing proline substitutions designed to preserve the antigenically relevant prefusion conformation. Expression is time-limited, and the absence of viral replication markedly constrains systemic exposure compared with natural infection. Although intramuscular injection is intended to favour local antigen expression, preclinical biodistribution studies indicate that lipid nanoparticles and vaccine mRNA are not exclusively confined to the injection site and draining lymph nodes. Importantly, however, biodistribution of the delivery platform or mRNA should not be considered equivalent to systemic distribution of biologically active spike protein.

## 5. Cardiac and Hematological Toxicities of the Spike Protein

Cardiovascular manifestations associated with COVID-19 include myocarditis, endothelial dysfunction, arrhythmias, and thromboembolic events [15,16]. These complications are multifactorial and cannot be attributed exclusively to spike protein; however, experimental evidence suggests that spike may contribute to several relevant pathogenic pathways, particularly endothelial dysfunction and thrombo-inflammation. Spike-mediated ACE2 downregulation, endothelial activation, complement engagement, and platelet–endothelial interactions have all been implicated [9].

In the hematological compartment, spike-related signaling has been linked experimentally to endothelial-dependent platelet activation and complement amplification. At the same time, COVID-19-associated immune-thrombosis more broadly involves platelet hyperreactivity, neutrophil extracellular trap (NET) formation, complement activation, and dysregulated coagulation. These pathways interact bidirectionally: complement activation promotes endothelial and platelet activation, activated platelets facilitate NET formation, and NETs provide a procoagulant scaffold that further amplifies thrombin generation and microvascular thrombosis [17]. Although some of these thrombo-inflammatory pathways overlap with mechanisms proposed in vaccine-associated adverse events, they should be distinguished from vaccine-induced immune thrombotic thrombocytopenia (VITT), in which anti-PF4 antibody-mediated platelet activation represents the central pathogenic mechanism, as discussed below [17].

These mechanisms may be conceptualized as an interconnected, amplifying cascade rather than as independent pathways. Initial spike–host interactions may involve ACE2-dependent signaling and/or integrin-mediated endothelial activation, resulting in endothelial dysfunction, increased permeability, and a proinflammatory and procoagulant phenotype. Endothelial activation may subsequently facilitate complement engagement and platelet–endothelial interactions, while activated platelets and complement can promote neutrophil activation and NET formation. NETs, in turn, provide a procoagulant scaffold that enhances thrombin generation and microvascular thrombosis, creating a feed-forward thrombo-inflammatory loop [7,9,17]. Importantly, this sequence represents a proposed mechanistic hierarchy rather than a firmly established linear pathway; several components may occur in parallel and may be strongly influenced by viral replication, systemic inflammation, and host susceptibility.

## 6. Molecular and Functional Differences Between Viral Infection–Induced and Vaccine-Induced Spike Protein

Key distinctions exist between infection-derived and vaccine-derived spike proteins. Natural infection produces spike in the context of active viral replication, inflammatory amplification, and tissue injury. In contrast, mRNA vaccination induces transient expression of a prefusion-stabilized spike antigen without viral propagation [4,13]. Differences in molecular configuration, cellular localization, magnitude and duration of antigen exposure, and the surrounding immune and inflammatory context may contribute to the markedly different biological and clinical consequences of infection and vaccination [18].

At the structural level, the prefusion-stabilizing proline substitutions used in mRNA vaccine-encoded spike restrict the large conformational rearrangements of the S2 subunit that normally accompany transition toward the post-fusion state. Native viral spike, in contrast, remains conformationally dynamic: receptor-binding domains alternate between ‘down’ and ACE2-accessible ‘up’ states, and receptor engagement together with proteolytic activation promotes subsequent fusion-associated rearrangements. Importantly, prefusion stabilization does not abolish ACE2 recognition, but it can modify receptor-induced conformational transitions and the accessibility of conformationally sensitive or cryptic epitopes. Thus, differences in conformational dynamics and epitope exposure, rather than simply the presence or absence of ACE2 binding, may influence the immunological and biological properties of vaccine-encoded versus infection-derived spike. These structural differences further caution against assuming biological equivalence between spike exposure in infection and vaccination [3,19].

Importantly, the presence of spike protein in both settings does not imply equivalent biological exposure or pathogenic potential. During infection, spike expression occurs alongside replicating virus, additional viral proteins, pathogen-associated molecular patterns, and extensive innate immune activation, whereas vaccination lacks viral replication and the accompanying viral proteome. Moreover, the magnitude, anatomical distribution, and duration of antigen exposure differ substantially between these settings. Therefore, mechanistic observations derived from infection-associated spike exposure should not be directly extrapolated to vaccine-derived spike without consideration of dose, molecular form, tissue context, and duration of exposure.

## 7. Spike Protein Persistence as a Potential Mechanism in Long-COVID

Long-COVID encompasses a heterogeneous spectrum of symptoms persisting for months after acute infection, including fatigue, dyspnea, cognitive dysfunction, dysautonomia, and thrombotic complications [20]. Among several proposed mechanisms, persistence of viral antigens or residual viral reservoirs has emerged as a potential contributor to Long-COVID, possibly sustaining chronic immune activation, endothelial dysfunction, and tissue-specific inflammatory responses.

Using an ultrasensitive antigen-detection platform, Swank et al. identified circulating full-length spike protein in a subset of individuals with post-acute sequelae of COVID-19, in some cases more than 12 months after the initial infection [10]. Subsequent studies have provided additional evidence that SARS-CoV-2 antigens can persist in plasma during the post-acute phase, with one or more viral antigens detected in approximately one-quarter of participants at one or more time points up to 14 months after infection [21]. Evidence of persistent viral material in extrapulmonary tissues further supports the possibility that tissue reservoirs may contribute to sustained antigen exposure and chronic host responses in a subset of individuals [22].

These findings should nevertheless be interpreted in the context of the analytical limitations of ultrasensitive antigen assays. Single-molecule array platforms enable detection of circulating viral antigens at very low concentrations; however, assay performance depends on antigen-specific limits of detection, antibody affinity, epitope accessibility, and the molecular form of the circulating antigen. Antigen–antibody complex formation may further influence detectability, and measurements obtained across different assay configurations are not necessarily directly comparable. Therefore, detection of ultralow circulating spike concentrations should not, in isolation, be interpreted as evidence of biologically active antigen or a causal role in persistent symptoms. Further assay standardization and independent validation are needed before circulating spike measurements can be considered clinically interpretable biomarkers.

However, antigen persistence is not detectable in all individuals with Long-COVID, and its presence does not by itself establish a causal relationship with persistent symptoms. Long-COVID is therefore likely to represent a heterogeneous condition involving multiple, potentially overlapping mechanisms rather than a single spike-driven process.

The observation that persistent circulating viral antigens are detectable only in a subset of individuals suggests that host-related factors may influence antigen persistence, clearance, and downstream biological responses. Potential modifiers include genetic background, HLA-dependent antigen presentation, pre-existing or infection-induced autoantibodies, the magnitude and coordination of antiviral immune responses, age, comorbidities, and the severity of the initial infection. HLA variation could theoretically influence viral antigen processing and T-cell recognition; however, specific HLA haplotypes have not yet been consistently linked to persistent spike antigenemia or Long-COVID and should therefore be considered candidate susceptibility factors rather than established determinants. Similarly, altered autoantibody profiles and persistent innate and adaptive immune dysregulation have been described in subsets of patients with Long-COVID, although their relationship to persistent spike antigen remains incompletely defined. These observations support a host–pathogen interaction model in which the biological consequences of persistent viral antigens may depend not only on antigen burden and duration but also on individual immune susceptibility [23,24].

## 8. Potential Biological Effects of Vaccine-Derived Spike Protein

The term ‘spikeopathy’ has been proposed as a conceptual framework to describe biological or pathological effects potentially attributable to spike protein exposure rather than to the complete virus [25]. Human studies have demonstrated transient circulating vaccine-derived spike antigen in some recipients, and free full-length spike has been detected in individuals with post-mRNA-vaccine myocarditis [26]. However, these observations establish an association rather than direct causality, and circulating spike may represent either a pathogenic mediator or a biomarker of altered antigen processing or immune dysregulation.

Vaccine mRNA and spike antigen have also been detected in draining lymph node germinal centers for several weeks following mRNA vaccination [18]. Importantly, such local antigen persistence should not be equated with pathological systemic persistence, as prolonged antigen availability within germinal centers may contribute to the maturation of adaptive immune responses.

Thus, while biological activity of vaccine-derived spike is experimentally plausible and antigen persistence has been demonstrated in specific anatomical and clinical contexts, evidence that these phenomena constitute a distinct systemic ‘spikeopathy’ remains insufficient. Mechanistic investigation of rare vaccine-associated adverse events may nevertheless provide important insights into host susceptibility, antigen processing, and immune responses.

Accordingly, extension of the ‘Long-Spike’ framework to post-vaccination conditions should be regarded as particularly speculative. Current evidence does not establish persistent vaccine-derived spike as a causal mechanism for chronic post-vaccination manifestations, and mechanistic observations from SARS-CoV-2 infection should not be directly extrapolated to the substantially different biological context of vaccination.

## 9. Vaccine-Induced Immune Thrombotic Thrombocytopenia and the Potential Role of Spike Protein

Vaccine-induced immune thrombotic thrombocytopenia is a rare but severe prothrombotic disorder characterized by thrombosis at unusual sites and thrombocytopenia, predominantly associated with adenoviral vector vaccines [27]. The central pathogenic feature is the development of high-titer IgG antibodies against platelet factor 4 (PF4), which form immune complexes that activate platelets via FcγRIIa receptors. This process promotes intense platelet activation, thrombin generation, and platelet consumption, thereby producing the characteristic combination of thrombosis and thrombocytopenia [28].

The precise trigger for anti-PF4 antibody formation remains incompletely understood. Several mechanisms have been proposed, including interactions among PF4, vaccine constituents, adenoviral vector components, inflammatory mediators, and host susceptibility factors [29]. Although spike protein has also been proposed as a potential contributor through endothelial activation or immune priming, direct evidence that spike itself initiates the anti-PF4 immune response characteristic of VITT remains insufficient. Accordingly, VITT provides an important example in which temporal association with spike-encoding vaccination should not be interpreted as evidence of direct spike-mediated causality; rather, the available evidence supports a distinct anti-PF4 immune thrombotic mechanism (Table 1).

## 10. Sticking the Pieces Together: The ‘Long-Spike’ Hypothesis

Integrating emerging evidence from studies of Long-COVID, persistent viral antigens, endothelial dysfunction, and immunothrombosis suggests that persistent or dysregulated exposure to spike-related antigens may contribute to chronic multisystem manifestations in a subset of susceptible individuals. On this basis, we propose the term “Long-Spike” as a hypothesis-generating conceptual framework rather than a defined clinical syndrome.

**Figure 1:**
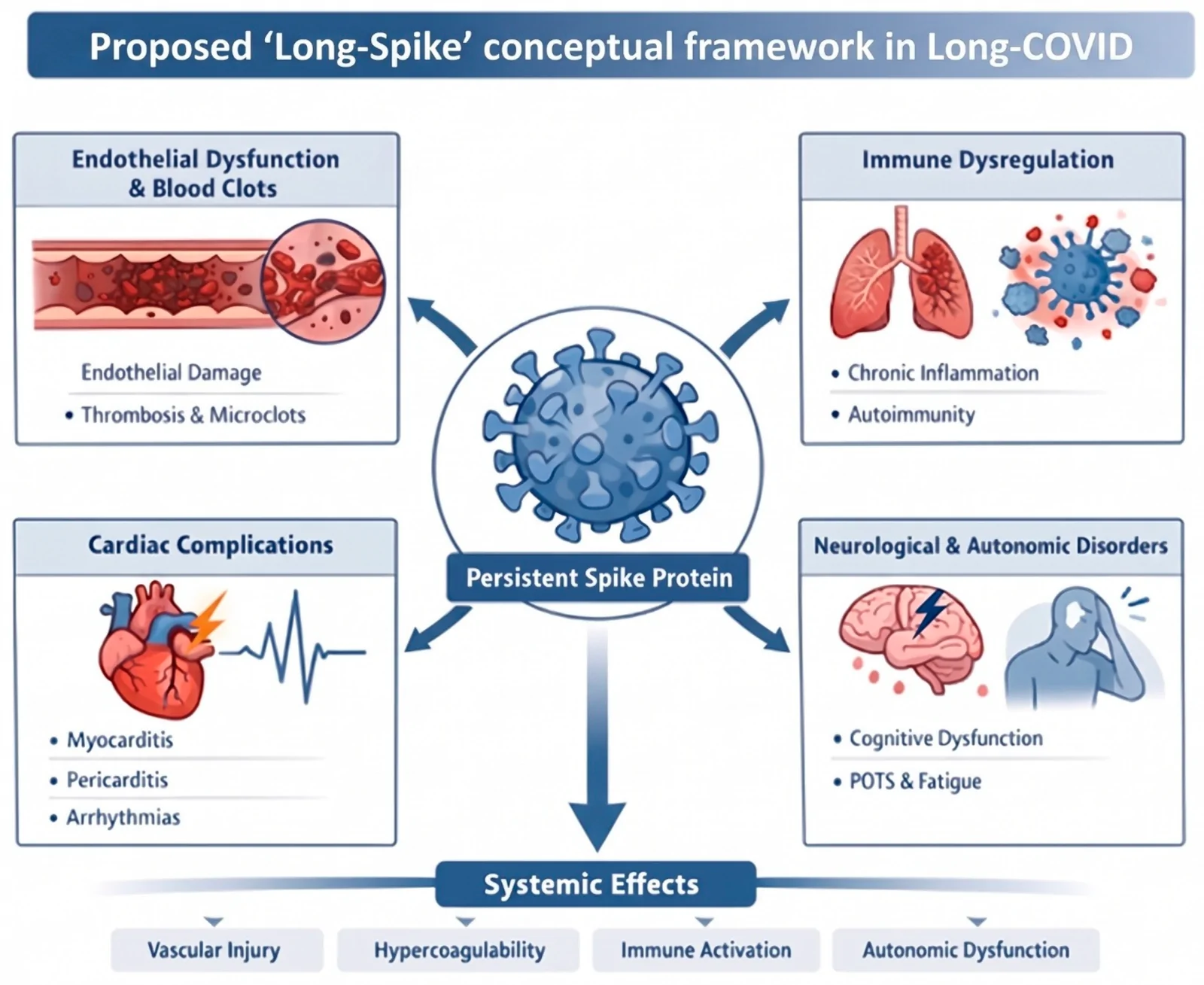
Proposed ‘Long-Spike’ conceptual framework in Long-COVID. Schematic illustration depicting persistent or dysregulated SARS-CoV-2 spike protein exposure as a central node linking endothelial dysfunction, immune dysregulation, platelet activation, microvascular thrombosis, autonomic imbalance, and organ-specific sequelae (cardiac, hematological, and neurological). The figure contrasts acute infection–driven spike exposure with post-acute persistence and highlights modifiers, including host susceptibility, comorbidities, and immune responses.

Within this framework, persistent or biologically active spike-related antigens could sustain interconnected processes including endothelial activation, platelet hyperreactivity, complement activation, immune dysregulation, and microvascular dysfunction, thereby contributing to cardiovascular, hematological, neurological, or autonomic manifestations (Figure 1). Importantly, this framework does not imply that infection-associated Long-COVID and rare vaccine-associated adverse events represent the same clinical entity or share equivalent mechanisms. Natural infection involves viral replication, multiple viral antigens, and a substantially greater inflammatory burden, whereas vaccination represents a fundamentally different biological exposure.

Rather, the potential conceptual overlap lies in whether prolonged or dysregulated spike antigen exposure under markedly different biological conditions may contribute to adverse outcomes in susceptible individuals. Evidence supporting such a mechanism is substantially stronger in post-infectious conditions than in post-vaccination syndromes, where causality remains largely unestablished.

These distinct clinical phenotypes should therefore not be viewed as consequences of a common spike-dependent threshold. In acute COVID-19, thrombo-inflammatory complications arise within the broader context of viral replication, systemic inflammation, endothelial injury, and coagulation activation, whereas Long-COVID is a heterogeneous post-acute condition in which persistent viral antigens may represent only one of several contributing mechanisms. VITT is mechanistically distinct, being primarily driven by anti-PF4 antibody-mediated platelet activation rather than established direct spike toxicity. Thus, differences in exposure profile and host response, rather than a single unifying spike-mediated mechanism, are likely to determine the biological and clinical consequences observed across these settings.

Testing the Long-Spike hypothesis will require prospective studies combining ultrasensitive antigen measurements with tissue-based analyses, immunophenotyping, endothelial and coagulation biomarkers, and longitudinal clinical phenotyping. Demonstrating a temporal and dose–response relationship between persistent spike antigenemia and specific clinical phenotypes, together with improvement following antigen clearance, would provide substantially stronger evidence for causality.

## 11. Conclusions and Future Perspectives

The SARS-CoV-2 spike protein occupies a central position at the intersection of virology, immunology, cardiology, and hematology. While spike-directed immune responses are central to protective immunity following infection and vaccination, accumulating experimental evidence indicates that spike protein itself may exert biological effects on endothelial, inflammatory, and thrombotic pathways under specific conditions. However, the strength of evidence differs substantially across clinical contexts. Evidence for persistent viral antigens and tissue reservoirs is increasingly recognized in subsets of patients following SARS-CoV-2 infection. In contrast, evidence linking persistent vaccine-derived spike to chronic clinical syndromes remains considerably more limited and does not currently establish causality.

Accordingly, the “Long-Spike” hypothesis proposed in this review should be regarded as a hypothesis-generating framework that integrates emerging observations rather than as a defined clinical syndrome or an established causal mechanism. Future research priorities include precise mapping of spike biodistribution and persistence, differentiation between circulating antigen and tissue reservoirs, identification of host susceptibility factors, and determination of whether persistent antigenemia correlates temporally and quantitatively with cardiovascular, hematological, neurological, or autonomic phenotypes.

Several practical implications follow from these knowledge gaps. For clinicians, detection of circulating spike or other viral antigens should currently be interpreted as an investigational finding rather than a validated diagnostic biomarker or an indication for targeted therapy, and evaluation of persistent symptoms should continue to follow established clinical pathways. For clinical trialists, prospective studies should incorporate standardized ultrasensitive antigen assays, predefined sampling intervals, appropriate post-infection and post-vaccination control groups, and parallel assessment of tissue reservoirs, immune phenotypes, endothelial and coagulation biomarkers, and clinically adjudicated outcomes. Such studies should also test temporal and dose–response relationships rather than relying solely on cross-sectional associations. For regulators and pharmacovigilance systems, infection-associated complications and rare vaccine-associated adverse events should be analyzed and communicated separately, with standardized case definitions and careful assessment of temporality, background incidence, and alternative mechanisms before causal attribution. These approaches may help distinguish biologically informative associations from clinically actionable causal relationships.

Maintaining scientific rigor, transparent reporting, and proportional risk communication remains essential. Carefully designed prospective studies that distinguish association from causation and directly compare infection-derived and vaccine-derived spike exposure will be required to determine the clinical relevance of persistent spike-related antigens and to refine future preventive and therapeutic strategies.

## Figures and Tables

**Table 1:** Cardio-hematological manifestations and proposed mechanisms across distinct SARS-CoV-2 infection- and vaccination-related clinical contexts.

Domain	Clinical Manifestation	Clinical/Exposure Context	Proposed Mechanism	Relationship to Spike
Cardiovascular	Myocardial injury/Myocarditis	Acute COVID-19	Viral infection, systemic inflammation, immune activation, and endothelial injury	Spike may contribute to endothelial and inflammatory signaling, but the clinical phenotype is multifactorial
Cardiovascular	Myocarditis	Rare post-mRNA-vaccination adverse event	Immune-mediated mechanisms; pathogenesis remains incompletely defined	Circulating free spike has been reported in affected individuals, but causalty has not been established
Cardiovascular	Endothelial dysfunction	Acute COVID-19	Viral replication, systemic inflammation; RAAS dysregulation, complement activation	Experimental evidence supports potential contributors from spike-ACE2 and integrin-mediated signaling
Cardiovascular/Multisystem	Persistent endothelial or microvascular dysfunction	Long-COVID/post-acute infection	Persistent viral reservoirs or antigens, immune dysregulation, and chronic inflammation	Persistent spike-related antigenemia is reported in a subset of patients; its causal significance remains uncertain
Hematological	Immunothrombosis/micro- and macrovascular thrombosis	Acute/severe COVID-19	Platelet activation, NET formation, complement activation, endothelial injury, and coagulation dysregulation	Spike-related signaling may contribute but represents only one component of a multifactorial process
Hematological	Thrombosis with thrombocytopenia	VITT after adenoviral-vector vaccination	Anti-PF4 IgG-mediated platelet activation through FcyRIIa	Direct spike-mediated causalty is not established; VITT represents a distinct anti-PF4 immune thrombotic mechanism
Autonomic/Neurological	Dysautonomia/POTS-like manifestations	Long-COVID/post-acute infection	Neuroimmune dysregulation and possible microvascular dysfunction	A direct causal role of persistent spike has not been established

ACE: Angiotensin converting enzyme; POTS: Postural orthostatic tachycardia syndrome; RAAS: Renin angiotensin aldosterone system; VITT: Vaccine-induced immune thrombotic thrombocytopenia. The inclusion of these conditions within the same table is intended for mechanistic comparison and does not imply a shared spike-mediated pathophysiology. In particular, VITT is a distinct anti-PF4 antibody-mediated disorder, and direct spike-mediated causality has not been established.

## Data Availability

No new data were created or analyzed in this study. Data sharing is not applicable to this article.
